# Drug-loaded nanoparticles for cancer therapy: A high-throughput multicellular agent-based modeling study

**DOI:** 10.1016/j.jtbi.2025.112266

**Published:** 2025-09-18

**Authors:** Yafei Wang, John Metzcar, Elmar Bucher, Heber L. Rocha, Vikram Jadhao, Randy Heiland, Hermann B. Frieboes, Paul Macklin

**Affiliations:** a Department of Intelligent Systems Engineering, Indiana University, Bloomington, Indiana, 47408, USA; b Department of Bioengineering, University of Louisville, Louisville, Kentucky, 40292, USA

**Keywords:** Agent-based modeling, Anticancer drug-loaded nanoparticles, Cancer nanotherapy, Nanoparticle inheritance

## Abstract

Interactions between biological systems and engineered nanomaterials have become an important area of study due to their application in medicine. In particular, the opportunity to apply nanomaterials for cancer diagnosis and treatment presents a challenge due to the complex biology of this disease, which spans multiple time and spatial scales. A systems-level analysis from mathematical modeling and computational simulation to explore the interactions between anticancer drug-loaded nanoparticles (NPs), cells, and tissues, and the associated system parameters and patient response would be of benefit. Although a number of models have explored these interactions in the past, few have focused on simulating individual cell-NP interactions. This study develops a multicellular agent-based model of cancer nanotherapy that simulates NP internalization, drug release within the cell cytoplasm, inheritance of NPs by daughter cells at cell division, cell pharmacodynamic response to intracellular drug levels, and overall drug effect on tumor growth. A large-scale parallel computational framework is used to investigate the impact of pharmacokinetic design parameters (NP internalization rate, NP decay rate, anticancer drug release rate) and therapeutic strategies (NP doses and injection frequency) on tumor growth. In particular, through the exploration of NP inheritance at cell division, the results indicate that cancer treatment may be improved when NPs are inherited at cell division for *cytotoxic* chemotherapy. Moreover, smaller dose of *cytostatic* chemotherapy may also improve inhibition of tumor growth when cell division is not completely inhibited. This work suggests that slow delivery by heritable NPs can drive new dimensions of nanotherapy design for more sustained therapeutic response.

## 1. Introduction

There is an enormous interest in the application of nanomaterials in medicine for therapy and diagnosis as well as understanding the associated nanomaterial-biological system interactions. Engineered nanomaterials have received particular attention due to the opportunities they offer with tailorable functionalities (e.g., desired shape, size, and surface compositions) to enable target-specific drug delivery with high efficiency and low side-effects (Nel et al., 2009; Pelaz et al., 2013). Sun et al. gave a detailed review of engineered nanoparticles (NPs) for drug delivery in cancer therapy, covering different anticancer drugs, methods of controlled release, NP and drug delivery, as well as case studies (Sun et al., 2014). Shi et al. summarized recent work on cancer nanomedicine and novel engineering methods used to improve the understanding of tumor biology and nano-biological interactions to develop more effective nanotherapeutics for cancer treatment (Shi et al., 2017). NPs have several advantages as therapeutic agent carriers in cancer therapy: (1) NPs can increase therapeutic agents’ solubility and circulation half-life as well as improve bio-distribution and permeability (Sun et al., 2014; Shi et al., 2017); (2) NPs can increase tumor cell targeting specificity through selective binding to overexpressed cell surface receptors through the addition of targeting ligands (e.g., peptides, antibodies, and nucleic acids) to the NP surface, reducing collateral damage to healthy cells (Chow et al., 2011); (3) multiple types of therapeutic agents can be delivered within the same NP, potentially reducing cancer drug-resistance by adjusting the ratio of different types of agents (Miao et al., 2017; Dai et al., 2017); (4) NPs may improve cancer immunotherapy through the development of synthetic vaccines by incorporating other molecules such as DNA, small interfering RNA (siRNA), messenger RNA (mRNA) and protein (Shi et al., 2017; Riley et al., 2019); (5) NPs can be engineered to allow controlled release of encapsulated therapeutic agents based on particular physiological or external stimuli, e.g., pH, enzymes, temperature, or electromagnetic radiation (Dogra et al., 2019).

Despite these advantages, NPs face a formidable journey from injection to effective intracellular action, navigating abnormal physical and physiological properties that present delivery barriers (Chauhan et al., 2011). These barriers include circulation in blood vasculature, uptake by immune cells, extravasation from capillaries, accumulation at the desired location, diffusion through extracellular space, endocytosis by cells, endosomal escape, intracellular localization and final action (Nichols and Bae, 2012; Stillman et al., 2020). Dogra et al. (2019) and Stillman et al. (2020) discussed three broad phases of NP transport from injection site to action site (tumor cells): vascular, transvascular, and interstitial. They also discussed NP-receptor binding, endocytosis, and intracellular NP delivery. See recent reviews Jain and Stylianopoulos (2010), Chauhan et al. (2011) and Barua and Mitragotri (2014) for further background biology.

Mathematical modeling of cancer nanotherapy could be beneficial for exploring NP-loaded anticancer treatments affected by cell and tissue interactions. Simulations could help efficiently investigate nanomedicine design parameters to maximize cytotoxic effect while reducing systemic toxicity. Prior mathematical models of NPs traveling from injection site to action site have investigated NP transport through the vasculature (Sefidgar et al., 2015), extravasation (Shah et al., 2018), tissue penetration (Hauert et al., 2013; Wu et al., 2014), endocytosis (Liu et al., 2010, 2011; Huang et al., 2013; Li et al., 2015) and intracellular trafficking. NPs may travel through different intracellular compartments, such as the cytoplasm, mitochondria, nucleus and lysosomes after internalization (Barua and Mitragotri, 2014). There is limited prior *in silico* modeling that addresses intracellular transport at this scale (Stillman et al., 2020). A detailed review of *in silico* modeling of cancer nanomedicine, across scales and transport barriers was presented in Stillman et al. (2020).

Prior computational modeling of cancer nanotherapy typically used ordinary differential equations (ODEs), partial differential equations (PDEs) and/or coupled models of PDEs, and agent-based models (ABMs). For example, Dogra et al. used an ODE-based physiologically based pharmacokinetic (PBPK) model to investigate the whole-body NP pharmacokinetics and the impact of key parameters on delivery efficiency, such as NP degradation rate, NP size and tumor blood viscosity (Dogra et al., 2020). More recently, Dogra et al. developed an ODE-based pharmacokinetic/pharmacodynamic (PK/PD) model to perform translational modeling (modeling bridging from preclinical to clinical development) for NP-mediated miRNA-22 therapy in triple negative breast cancer (Dogra et al., 2021). Such ODE-based PBPK, PK/PD and more complicated quantitative systems pharmacology models have been widely used to quantitatively explore NP transport, delivery into tumor cells, and drug effects, and enable investigation of dose-response relationships and optimization of treatment frequency for evaluations of preclinical/clinical trials. These models generally cannot account for tumor cell heterogeneity and spatial interactions. Beyond ODE approaches, PDEs and/or hybrid PDE/ABM approaches have also been employed in cancer nanotherapy research. For example, Frieboes and co-workers evaluated cancer nanotherapy that included multiscale effects in a continuum tumor tissue representation, including NP transport, drug release and effects on vascularized tumor growth dynamics (Frieboes et al., 2010; Curtis et al., 2015, 2016; Miller and Frieboes, 2019b,a; Chamseddine et al., 2020). These PDE and hybrid ABM/PDE models have advanced the modeling of tumor cell heterogeneity and spatial interactions (e.g., NP and released-drug distribution within tumor tissue as well as tumor cell response to the microenvironments). However, since these prior works modeled tumors via PDEs, they could not track NP internalization and drug release for individual cells.

In this work to model cancer nanotherapy, we use a hybrid discrete-continuum approach (Metzcar et al., 2019) to investigate internalized NPs in individual cells, heterogeneity of tumor cells, and spatial interactions at the cell-NP, cell-cell, and cell-tissue scales. The framework simulates discrete cell agents that model individual cancer cells, coupled with PDE representations of extracellular oxygen and NP concentration fields. In addition, each cell agent contains a system of ODEs to represent the population of internalized NPs across their drug release states, as well as the total amount of released drug. In an advance over prior models, within each cell, we model a *population* of internalized NPs including NP drug release states. This allows us to more accurately track NP history, providing a more nuanced view of drug release within the cytoplasm. Additionally, these NPs are heritable: when a cell divides, each daughter cell inherits half of the parent cell’s NP population. Through this generational inheritance, our model admits new classes of therapeutic approaches, where drug-loaded NPs can persist through multiple cancer cell generations, potentially enabling long-term cancer control.

## 2. Methods

### 2.1. Physicell: a multicellular simulation framework

In Ghaffarizadeh et al. (2018), Macklin and coworkers developed *PhysiCell*, a framework for off-lattice agent-based simulations in multicellular systems biology. In the framework, each cell agent has a phenotype with a hierarchically structured set of properties including state and rate parameters for cell cycling, cell death (apoptosis and necrosis), volume regulation (fluid and solid biomass, nuclear and cytoplasmic subvolumes), motility, cell-cell mechanical interactions, secretion/uptake, and intracellular pathway reactions. More recent additions to PhysiCell’s built-in phenotype include cell transformations, phagocytosis, and effector cell attacks. Each cell agent can sample the microenvironment (through BioFVM (Ghaffarizadeh et al., 2015), PhysiCell’s coupled diffusion solver), which is useful for modeling microenvironment-dependent triggers of standard cell processes. Each cell agent can have custom C++ rules assigned to model novel hypotheses (e.g., through rules of interpretable cell behavior (Johnson et al., 2025)), which we use here to model NP internalization dynamics, NP drug release, anticancer drug effect on cancer cell growth dynamics, and distribution of NPs to daughter cells at cell division. See Ghaffarizadeh et al. (2018) for full algorithmic detail, numerical testing, and a variety of examples. *PhysiCell* has been applied to a broad variety of multicellular system problems, such as oncolytic virus therapy, cancer immunology, tissue mechanics, infection dynamics and tissue damage, cancer mRNA vaccine treatments, cancer cell migration, extracellular matrix remodeling, and cellular fusion, among others (Jenner et al., 2022; Ozik et al., 2018; Rocha et al., 2024; Wang et al., 2021a; Getz et al., 2021; Wang, 2022; Rocha et al., 2021; Metzcar et al., 2025; Risner et al., 2020). See Metzcar et al. (2019) for detailed review of cell-based computational modeling in cancer biology.

**Fig. 1.**
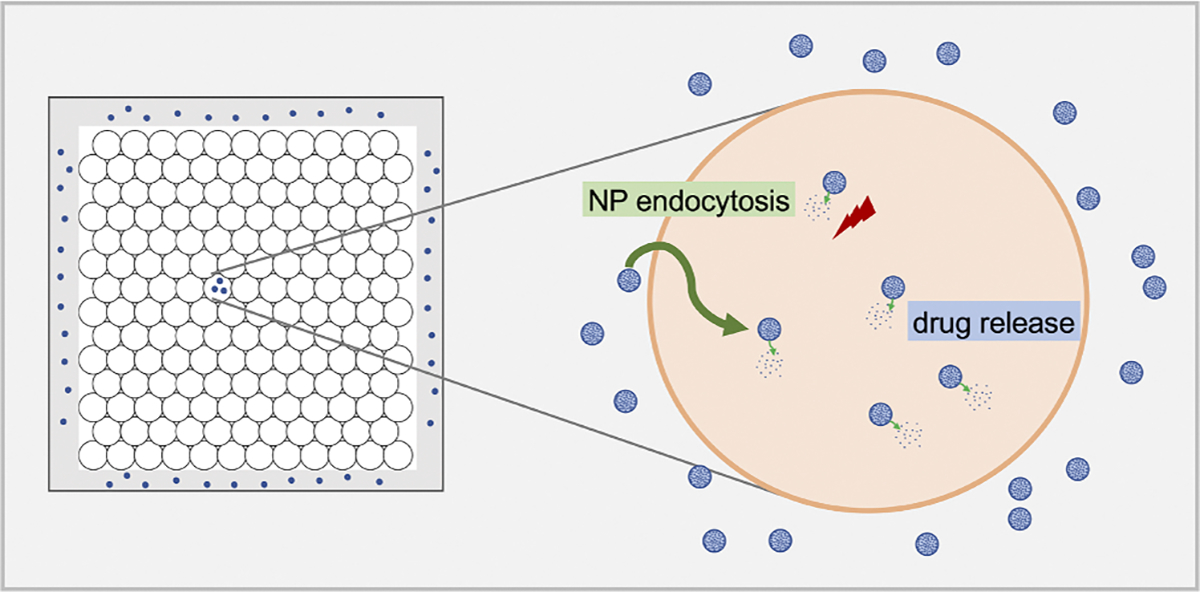
Schematic diagram of the overall mathematical modeling approach. A 2D tumor is initialized and NPs are released from the domain boundary, representing release from blood vessels. Extracellular NPs are modeled as a continuum using PDEs to simulate diffusion through the microenvironment. After NPs are internalized inside tumor cells via endocytosis, they start to release anticancer agents. Tumor cell phenotype (cycling and apoptosis) is impacted by the drug effects following rules defined in the model.

We used PhysiCell (Ghaffarizadeh et al., 2018) (version 1.7.1) to develop a multiscale ABM of cancer nanotherapy, and used it to investigate tumor cell growth and interactions with NP encapsulating anticancer drugs. The model includes modules of net NP internalization dynamics, drug release, NP inheritance and drug effect on tumor cell phenotype. The overall approach is summarized in Fig. 1. The source code is available at: https://github.com/MathCancer/PhysiCell-nanobio.

### 2.2. Cell-based model implementation details

We use the *Live* cell cycle model of *PhysiCell* (Ghaffarizadeh et al., 2018; Juarez et al., 2016), where live cells can divide into two live cells with birth rate b. Each cell can divide, apoptose, or necrose based upon user-defined functions. We use PhysiCell’s built-in cell mechanics model, which includes both repulsive and adhesive forces to govern cell-cell interactions, cellular motility, and viscous like-drag forces. Each cell’s position is updated based on the balance of these forces, assuming an inertialess regime where velocity is determined explicitly. We use the built-in oxygen-dependent proliferation and necrosis: notably, the “Live” cell cycle transition rate (b) increases with oxygen availability (σ) above a minimal hypoxic threshold, and the necrotic death rate ($r_{\text{nec}}$) increases below the threshold. See Eqs. (1) and (2).


(1) $$b(\sigma )=\overset{‾}{b}\cdot min\left\{1,max\left\{\left(\frac{\sigma \;-\;\sigma_{1}}{\sigma_{2}\;-\;\sigma_{1}}\right),0\right\}\right\}$$

(2) $$r_{\text{nec}}(\sigma )={\overset{‾}{r}}_{\text{nec}}\cdot min\left\{1,max\left\{\left(\frac{\sigma_{3}\;-\;\sigma }{\sigma_{3}\;-\;\sigma_{4}}\right),0\right\}\right\}$$

In Eqs. (1) and (2):

- $\overset{‾}{b}$: reference proliferation rate;
- $\sigma_{1}$: oxygen proliferation threshold. Oxygen below which the proliferation ceases;
- $\sigma_{2}$: oxygen proliferation saturation. Oxygen above which the proliferation rate is maximized;
- ${\overset{‾}{r}}_{\text{nec}}$: maximum necrosis rate;
- $\sigma_{3}$: oxygen necrosis threshold. Oxygen value at which necrosis starts;
- $\sigma_{4}$: oxygen necrosis maximum. Oxygen value at which necrosis rate reaches its maximum.

See the supplementary table and *PhysiCell* (Ghaffarizadeh et al., 2018) for further details and default parameter values, as well as recent experimentally-validated modeling (Rocha et al., 2021) that used this functional form.

### 2.3. Oxygen and nanoparticle diffusion

We use BioFVM (Ghaffarizadeh et al., 2015), PhysiCell’s integrated diffusion solver, to simulate diffusion of the model substrates (oxygen and NPs). BioFVM uses the following form:

(3) $$\begin{array}{l} \frac{\partial \rho }{\partial t}=D\nabla^{2}\rho -\lambda \rho +S\left(\rho^{*}-\rho \right)-U\rho \\ \;+\sum_{\left\{\text{cells}\;i\right\}}\delta \left(x-x_{i}\right)V_{i}\left[S_{i}\left(\rho_{i}^{*}-\rho \right)-U_{i}\rho \right], \end{array}$$

where ρ is the vector of diffusible substrates at point x with diffusion coefficients D, decay rates λ, bulk source rates S (which saturate at target densities $\rho^{*}$), and bulk uptake rates U. The final term simulates cell-based secretion for a collection of cells (indexed by i) with centers $x_{i}$, volumes $V_{i}$, cell secretion rates $S_{i}$, saturation densities $\rho_{i}^{i}$, and uptake rates $U_{i}.\;\delta \left(x-x_{i}\right)$ is the Dirac delta function. BioFVM can employ zero-flux (Neumann) and Dirichlet boundary conditions, or a combination of both (mixed), all at the substrate level. Of these available features, we use substrate diffusion, decay, cell uptake, and Dirichlet boundary conditions. See Ghaffarizadeh et al. (2015) for full details on the numerical method, implementation, and convergence testing for BioFVM. Note also that except where necessary for clarity, we suppress ρ’s dependence on x.

**Fig. 2.**
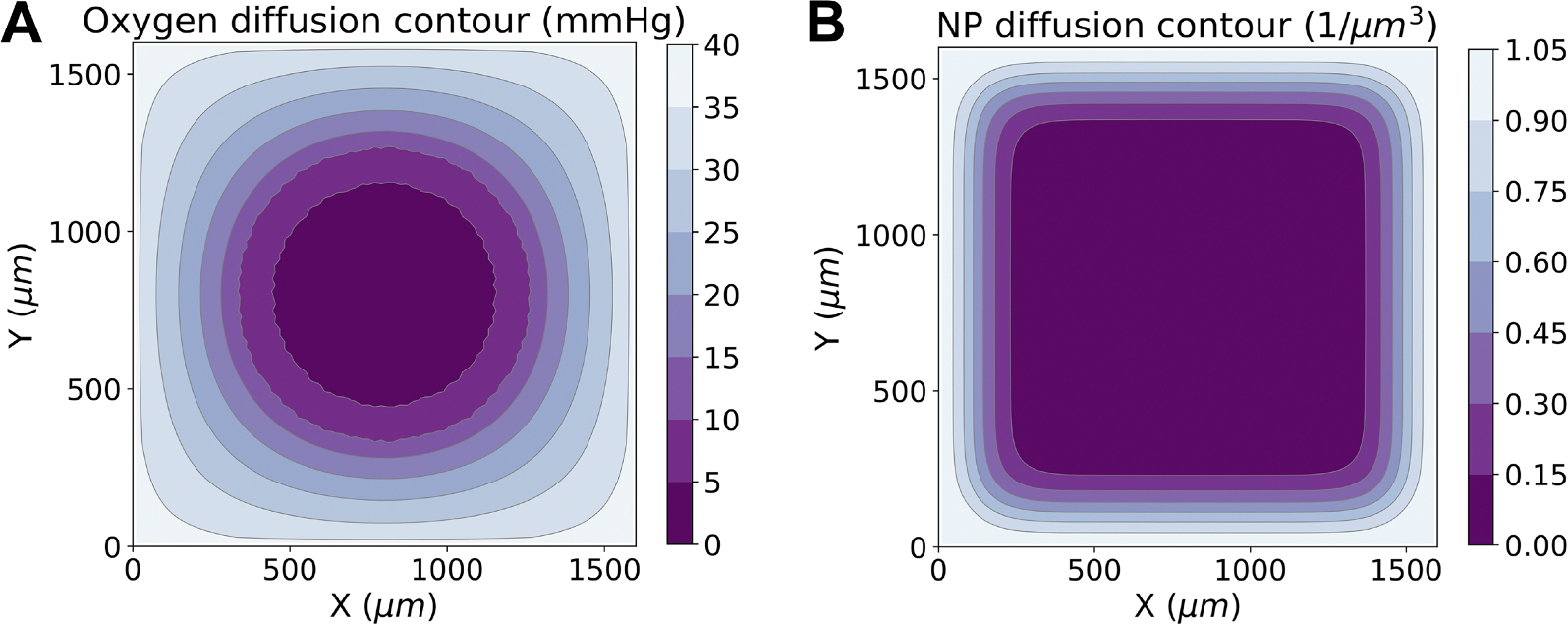
Oxygen (a) and NP (b) diffusion profiles at 24 simulated hours. Note, for illustrative purposes, we disabled cell cycling and death to preserve symmetry of the diffusion profile.

In the model, oxygen and NPs diffuse from the boundary to model a vascularized far-field condition. Note that NP diffusion is slower than oxygen diffusion ($6\;\mu m^{2}/min$ vs $1e5\;\mu m^{2}/min$). Fig. 2 shows oxygen and NP diffusion contours at 24 hours.

### 2.4. Nanopaticle internalization dynamics

For the *cell-level* model, we propose a mathematical model of internalization dynamics based on experimental results reported in the literature. Based on observations in Chithrani et al. (2006), the number of internalized NPs in a cell ($n_{I}$) has a maximum “saturated” value $n^{*}$ and the rate of internalization decreases as $n_{I}\to n^{*}$. In addition, it was noted that the rate of internalization increases with the NP tissue concentration $\rho_{NP}$ near and surrounding the cell. Accordingly, the rate of change for the number of NPs internalized in an individual celli is:

(4) $$\frac{dn_{I}}{dt}=r_{I}\left(1-\frac{n_{I}}{n^{*}}\right)V_{i}\rho_{NP}$$

where $r_{I}$ is the base NP internalization rate, and $V_{i}$ is celli’s volume.

For the *tissue-level* model, we adapt the conservation-based reaction-diffusion model from Ghaffarizadeh et al. (2015) as the governing equation of NP transport:

(5) $$\frac{\partial \rho_{NP}}{\partial t}=D\nabla^{2}\rho_{NP}-\lambda \rho_{NP}-\sum_{\text{cells}\;i}U_{i}V_{i}\delta \left(x-x_{i}\right)\rho_{NP}$$

where D is NP diffusion coefficient, λ is NP decay rate in the environment, $U_{i}$ is the NP uptake rate for $\text{cell}\;i,\;V_{i}$ is celli volume, $\delta \left(x-x_{i}\right)$ is Dirac delta function, and $x_{i}$ is the celli center.

The secretion of NPs is not simulated (or equivalently, only *net* uptake is modeled). We integrate over the domain Ω and simplify the result as follows (neglecting the effect of decay and diffusion by assuming short times):

(6) $$\frac{dn_{E}}{dt}\;=\frac{\partial }{\partial t}\int_{\Omega }\rho_{NP}dV=-\sum_{\text{cells}\;i}U_{i}V_{i}\int_{\Omega }\delta \left(x-x_{i}\right)\rho_{NP}dV\;\;=-\sum_{\text{cells}\;i}U_{i}V_{i}\rho_{NP}\left(x_{i}\right)$$

where $n_{E}$ is the total number of (non-internalized) NPs within a tissue domain Ω.

#### Matching the tissue-level and cell-level models:

Suppose we have a single cell and match the tissue-level equation to the cell-level equation, neglecting the effect of decay by assuming short times. To maintain conservation of NPs, we require:

(7) $$-\frac{dn_{E}}{dt}=\frac{dn_{I}}{dt}$$

(8) $$\Rightarrow U_{i}V_{i}\rho_{NP}\left(x_{i}\right)=r_{I}\left(1-\frac{n_{I}}{n^{*}}\right)V_{i}\rho_{NP}\left(x_{i}\right)$$

(9) $$\Rightarrow U_{i}=r_{I}\left(1-\frac{n_{I}}{n^{*}}\right)$$

Because we can relate changes in the extracellular NP concentration (via uptake) to changes in intracellular number of NPs, we can track NP internalization for individual cells.

### 2.5. Nanoparticle drug release

**Fig. 3.**
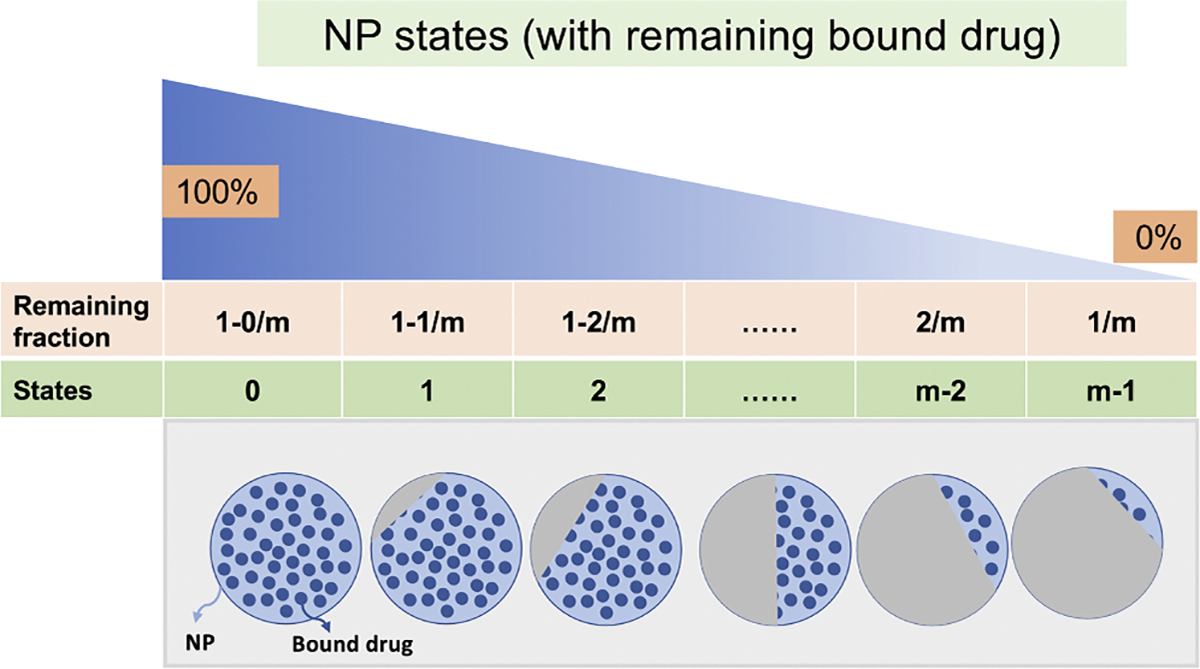
Schematic diagram of NP drug release states. In the model, each NP has one of m drug release states with the first state 0 corresponding to no drug released from the NP (100 % drug remaining) and the final state (m-1) corresponding to 1/m (so all drug would be released at next Δt, 0 % drug remaining). As drug is released from a NP, it transitions to a state with less remaining drug. This drug release is modeled via NP transitions from state to state, represented with the blue to white gradient (top) and decreasing amount of drug in the nanoparticle schematic (grey circles with blue drug). In the bottom diagram, the large circle indicates one NP and the small solid dots represents remaining bound drug.

Each NP arriving in a cell (with index i as above) individually releases drug, leading to a *population* of internalized NPs with heterogeneous drug release states. See Fig. 3 for overall diagram of drug release.

In the work, we propose an “age”-structured model of m possible states of drug release. If each NP arrives with $C^{*}$ bound drug, then for each 0≤j<m, define the $j^{\text{th}}$ population $n_{j}$ as containing $C_{j}$ bound drug and satisfying

(10) $$f_{j+1}C^{*}\leq C_{j}\leq f_{j}C^{*},$$

where $f_{j}=\left(1-\frac{j}{m}\right)$ for any 0≤j<m. For simplification in calculations below, we shall use $C_{j}\approx f_{j}C^{*}$; future work could use the improved midpoint approximation $C_{j}\approx \frac{1}{2}\left(f_{j+1}+f_{j}\right)C^{*}$.

If $n_{I}$ is the total amount of internalized NPs in the ($i^{\text{th}}$) cell, then notice that these populations satisfy

(11) $$n_{I}=\sum_{j=0}^{m-1}n_{j}.$$

Next, we seek a transition model as NPs release bound drug and transition between these release states, while also allowing for the possibility of internalized NP removal or “decay” (with rate $\lambda_{NP}$). If $\alpha_{j}$ is the transition rate of NPs from $n_{j}$ to $n_{j+1}$, then (using Eq. (8) for the arrival rate into $n_{0}$), we have

(12) $$\frac{dn_{0}}{dt}=r_{I}\left(1-\frac{n_{I}}{n^{*}}\right)V_{i}\rho_{NP}\left(x_{i}\right)-\alpha_{0}n_{0}-\lambda_{NP}n_{0}\;\;\vdots$$

(13) $$\frac{dn_{j}}{dt}=\alpha_{j-1}n_{j-1}-\alpha_{j}n_{j}-\lambda_{NP}n_{j}(1\leq j\leq m-2)\;\;\vdots$$

(14) $$\frac{dn_{m-1}}{dt}=\alpha_{m-2}n_{m-2}-\lambda_{NP}n_{m-1}.$$

To determine these transition rates $\alpha_{j}$, define the time $\tau_{j}=\frac{1}{\alpha_{j}}$ to be the spent in state j (in population $n_{j}$). To move from one state to the next, a NP must release $\frac{1}{m}C^{*}$ drug. Thus, if $r_{j}$ is the release rate for state j (with dimensions drug per time), then

(15) $$\frac{1}{m}C^{*}=r_{j}\tau_{j}⟹\alpha_{j}=\frac{1}{\tau_{j}}=\frac{m}{C^{*}}r_{j}.$$

We use the drug release rates $r_{j}$ to model the released drug C within the $i^{\text{th}}$ cell’s cytoplasm:

(16) $$\frac{dC}{dt}=\sum_{j=0}^{m-1}r_{j}n_{j}-\lambda_{drug}C$$

where $\lambda_{drug}$ is the degradation or decay rate of released drug.

Lastly, we seek a constitutive relation for $r_{j}$; in this paper, we consider the simple form:

(17) $$r_{j}=\beta C_{j}=\beta f_{j}C^{*}=\gamma_{1}f_{j},$$

where $\gamma_{1}=\beta C^{*}$ is a characteristic release rate and $r_{j}$ depends on the fraction of drug remaining in the nanoparticle.

**Fig. 4.**
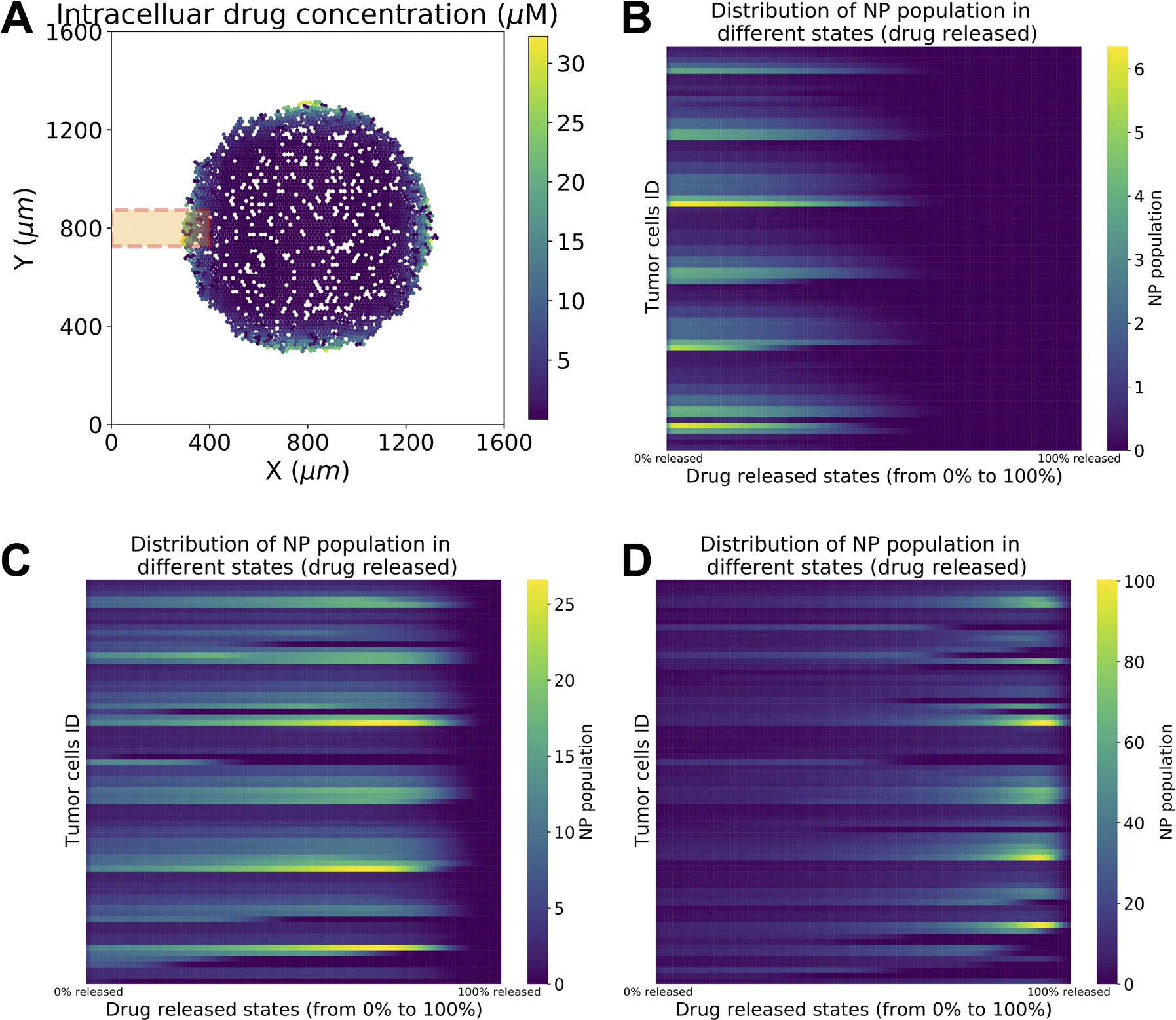
Distribution of NP drug release states, as tracked in individual cells. In (a), we plot the intracellular drug concentration across the whole tumor, early in treatment. The color bar indicates the number of NPs per cell. The NPs in the tumor cells in the orange-shaded 400 *μm* × 150 *μm* region are analyzed in (b–d). In (b–d), we visualize the distribution of drug release states for all cells in the highlighted region of (a) (shown in orange). The rows represent individual cells in the region of interest, while the columns show a heatmap of the NP population by the drug release state for each of these cells, from 0 % (left side) to 100 % (right side). As the simulation progresses, the overall NP populations shift to states of increased drug release. The color bars show the NP population count ($n_{j}$ from (11)) at a specific drug release state.

With the proposed model, we can track anticancer drug concentration and NP states inside individual cells. Fig. 4(b–d) presents the NP states at different simulation time points for the region of focus (orange box) in Fig. 4(a). Tumor heterogeneity in NP internalization and drug release can be clearly observed in the heterogeneous spectrum distribution of drug release states across tumor cells.

## 2.6. Nanoparticle inheritance

**Fig. 5.**
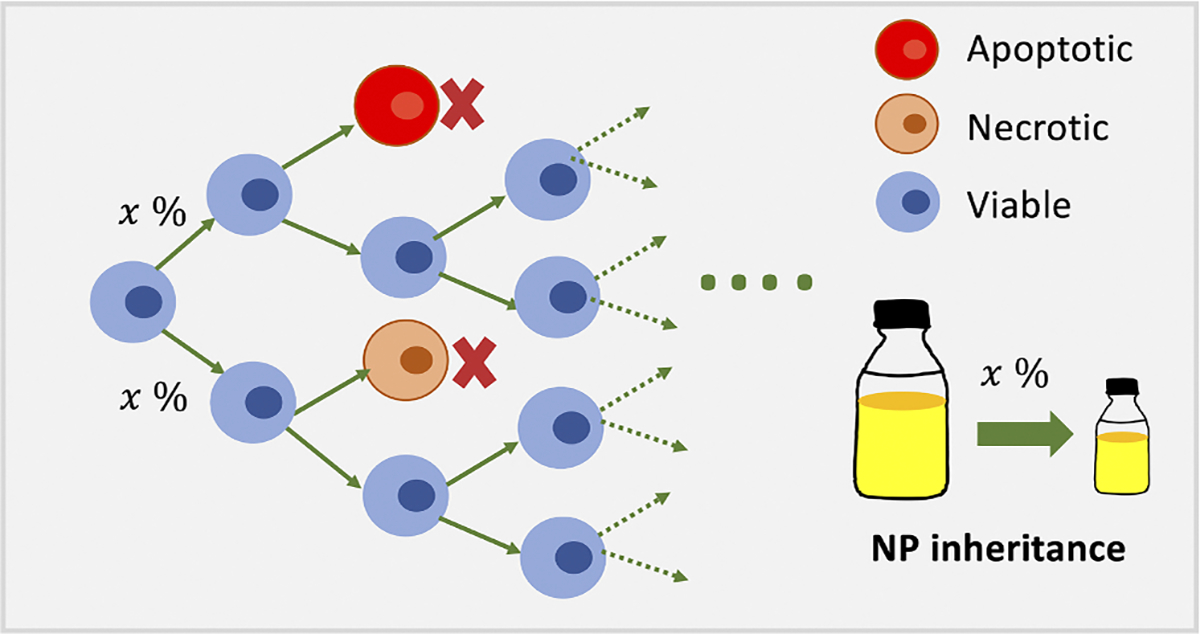
Schematic diagram of NP inheritance at cell division. In the model, tumor cells can divide, apoptose, or necrose (note that internalized NPs are removed from system if a cell dies). At cell division, the two daughter cells receive x% (with × running from 0 to 50) of the parent cell’s NPs.

When a cell divides, conservation of mass dictates that the number of NPs in the daughter cells equal those that were present in the parent cell; nonetheless, to the best knowledge of our knowledge, no other study to date has modeled and explored cancer nanotherapy where NPs are divided among the daughter cells at cell division. To simulate this effect, we enable cells to pass on up to 50 % of their current NP count to their daughter cells. See Fig. 5 for model diagram. We investigate in detail how NP inheritance impacts cancer treatments in Section 3.

### 2.7. Anticancer drug effect on cell phenotype pharmacodynamics

We calculate drug effect based on intracellular drug concentration using sigmoidal (Hill) response functions. See applications of Hill functions in pharmacological modeling and multicellular system modeling in Goutelle et al. (2008) and Johnson et al. (2025). Tumor cell phenotype (cycling or apoptosis rate, depending on the type of chemotherapeutic) is updated in the model by linearly interpolating the “base” phenotype (in the absence of drug) and maximal change in the phenotype under therapy, using the nonlinear drug effect E as the interpolating parameter. We use two common models for the drug effect E: Hill response functions, and area-under-the-curve (AUC) models.

#### Hill function model:

This “S-shaped” curve is given by

(18) $$E(t)=E_{max}\frac{c(t{)}^{n}}{{EC}_{50}^{n}\;+\;c(t{)}^{n}}$$

where ${EC}_{\text{50}}$ is the concentration at which the drug produces 50 % of the maximum effect, $E_{\text{max}}$ is the maximum drug effect, and n is the Hill power. The Hill function is used to model a drug that acts based upon its current concentration, rather than accumulated exposure time.

#### AUC model:

For other drugs, it may be more appropriate to model an effect based upon total (integrated) drug exposure; for such drugs, an AUC model captures this accumulated effect. The drug concentration c may be replaced by an integration over time-AUC (Area Under the Curve):

(19) $$AUC(t)=\int_{0}^{t}c(t)dt$$

(20) $$E(t)=E_{max}\frac{AUC(t{)}^{n}}{{EC}_{50}^{n}\;+\;AUC(t{)}^{n}}.$$

The cell’s phenotype (a vector of time-dependent parameters, including cycle entry, apoptosis, motility, mechanics, secretion and others) can vary continuously in time (Johnson et al., 2025) with “base” values $b_{0}(t)$ in the absence of drug effect E(t). In the presence of drug (E≠0), these phenotypic parameters are further perturbed towards maximally altered values $b_{\text{max}}$ as a linear interpolation of the nonlinear effect E, giving a current phenotype vector $b_{\text{phenotype}}\;(t)$:

(21) $$b_{\text{phenotype}}\;(t)=b_{0}+\left(b_{max}-b_{0}\right)\frac{E(t)}{E_{max}}.$$

(For parameters b unaffected by the drug, we set $b_{\text{max}}=b_{0}(t)$.) Please see Johnson et al. (2025) for more detailed and generalized discussion of varying cell phenotype with intracellular and extracellular signals including therapeutics as done in this model. This model framework can also model other drug effects such as impact on cellular uptake rate, secretion rate, mechanics, or motility. Refer to our cloud-hosted app for full model demonstration.

### 2.8. Computing and cloud-hosted model app

**Fig. 6.**
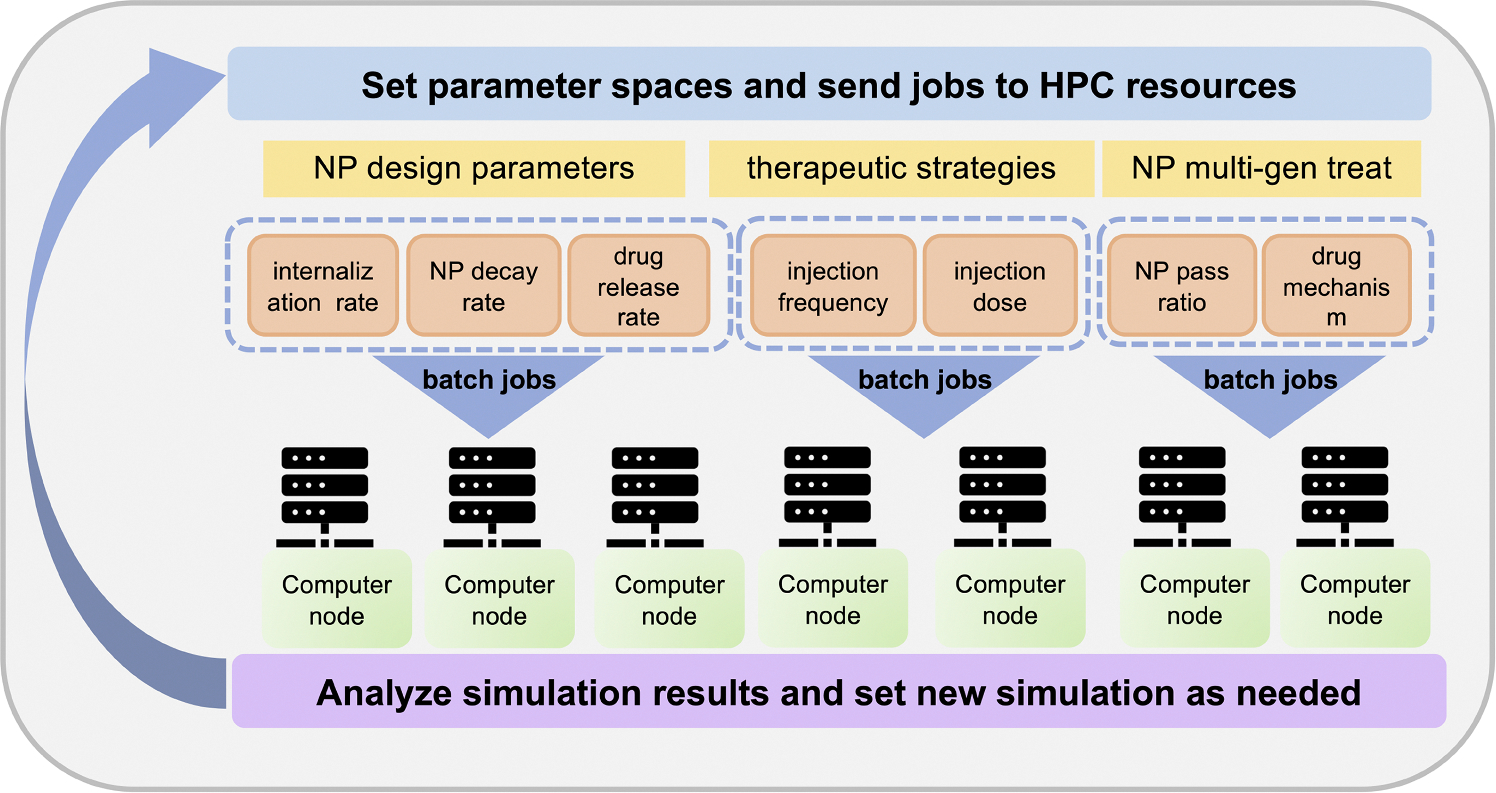
Schematic diagram of large-scale parameter exploration. In the investigation, parameter spaces are set and batch jobs are submitted to high performance computing (HPUC) resources for computing, collecting data and analyzing results.

Because the simulation model is stochastic, we ran 10 simulation replicates (each with a different random seed) for each parameter set. All simulation images are chosen from one representative replicate, and unless noted otherwise, all aggregate dynamical curves (e.g., viable cells) are reported as the mean and confidence interval (defined as ± one standard deviation) over all replicates. Simulation times were selected to be illustrative of model phenomena. Simulations were performed on the Big Red 3 supercomputer at Indiana University. See Fig. 6 for schematic diagram of large-scale parameters exploration for ABM framework). Each simulation was run on a single node using 24 threads. All jobs were submitted as a batch.

**Fig. 7.**
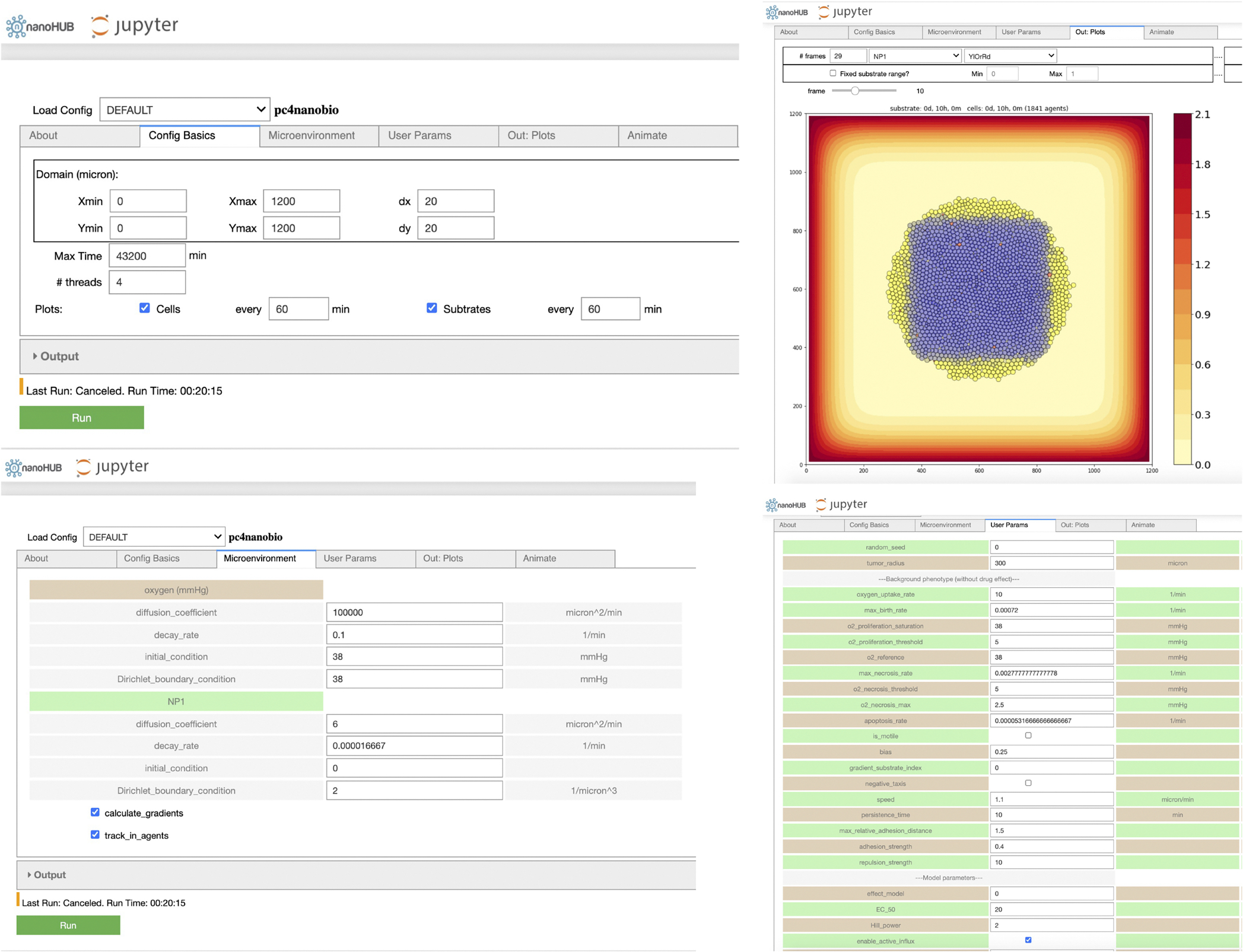
Cloud-hosted interactive model-*pc4nanobio* (version 1.0.0). User can freely access the online App to set up config parameters (e.g., domain size, substrates diffusion coefficients, custom data etc), simulate, and then plot simulation results. Interested readers can interactively run the model in a web browser at: https://nanohub.org/resources/pc4nanobio.

We used *xml2jupyter* (Heiland et al., 2019) to create a cloud-hosted version of this model: *pc4nanobio*; the model can be run interactively in a web browser at: https://nanohub.org/resources/pc4nanobio (Wang et al., 2022). Fig. 7 gives representative snapshots of the online model.

## 3. Results

After developing the ABM-based nanotherapy model, we investigated the interaction dynamics between anticancer drug-loaded NPs and cancer cells. We started by exploring how NP pharmacokinetic design parameters (internalization rate ($r_{I}$), drug release rate (γ’s) and decay rate ($\lambda_{NP}$)) impact tumor growth dynamics. Next, we used the framework to simulate tumor response with different therapeutic schedules (NP injection dose and frequency). Then, we focused the investigation on NP inheritance across cell generations. To ensure that our results can generalize, we investigated cytostatic drugs (those that slow cycle entry) and cytotoxic drugs (those that induce cell death).

### 3.1. Large-scale NP pharmacokinetic design space parameter exploration

**Fig. 8.**
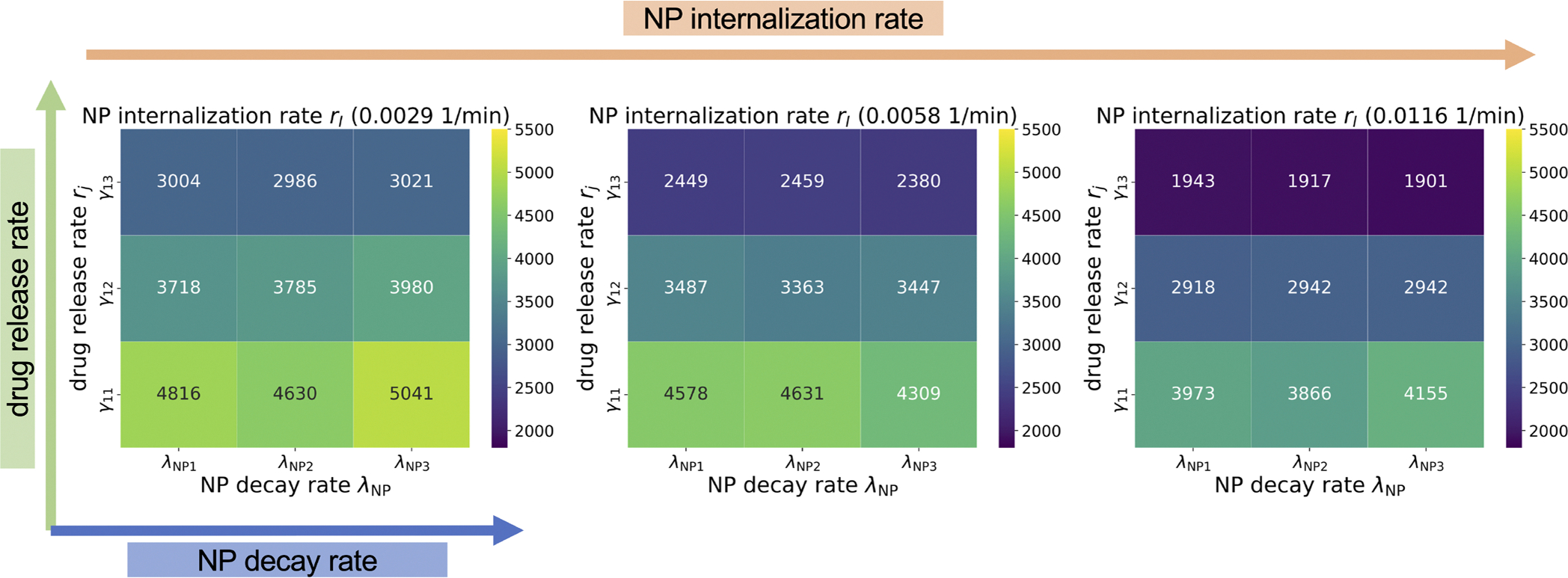
NP design parameters study: internalization rate, intracellular decay rate, drug release rate. We observe that the model is sensitive to the internalization and drug release rates, while it is relatively insensitive to the intracellular decay rate. This is especially apparent in the case of faster drug release (top rows of each square), because NPs release most of their drug load prior to their decay. The heatmap indicates the number of viable cells at 30 days post treatment. Each reported value is the mean of 10 simulation replicates. For the parameter exploration: drug release rate $\left(r_{j}\right)$ was varied through adjusting $\gamma_{1}$, as $\gamma_{11}$ (0.02 *μ*mol/min), $\gamma_{12}$ (0.1 *μ*mol/min), $\gamma_{13}$ (∞ or immediately release); NP decay rate $\left(\lambda_{NP}\right)$ was varied as $\lambda_{NP1}$ (3.2090e-05 1/min), $\lambda_{NP2}$ (9.6270e-05 1/min), $\lambda_{NP3}$ (4.8135e-04 1/min), which represents the half-life time as 15 days, 5 days and 1 day respectively; and NP internalization rate ($r_{I}$) was varied as low (0.0029 1/min), medium (0.0058 1/min), and high (0.0116 1/min).

**Fig. 9.**
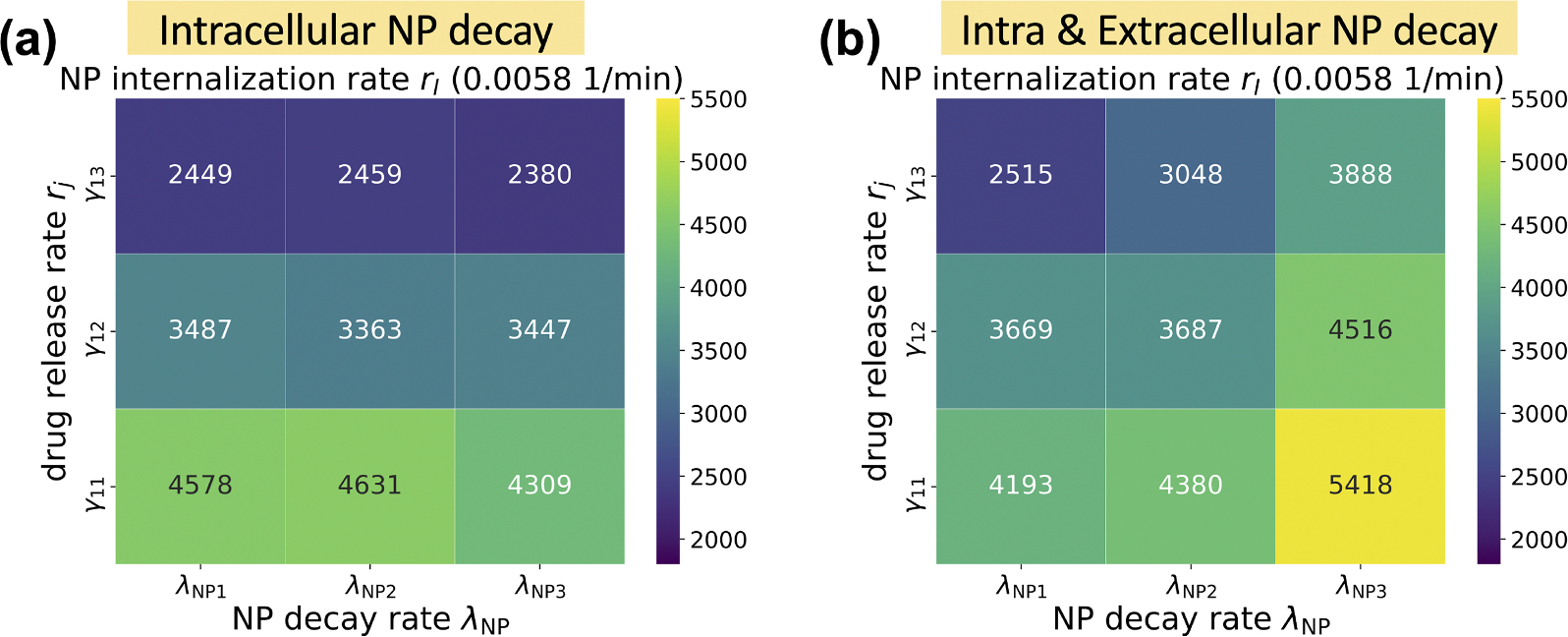
NP exploration: intracellular and extracellular decay rates of NPs. We observe that treatment efficacy was most impacted by the drug release rate in all scenarios, but rapid extracellular NP decay (1.6045e-5 1/min or half-life as 30 days) substantially reduced treatment efficacy due to the reduction in NPs endocytosed by cells. The heatmap indicates the number of viable cells at 30 days post treatment with dark blue squares indicating most effective treatments (fewest remaining tumor cells). Each reported value is the mean of 10 simulation replicates. All simulations were done with an internalization rate is 0.0058 1/min. Drug release rate ($r_{j}$) was varied through adjusting $\gamma_{1}$, as $\gamma_{11}$ (0.02 *μ*mol/min), $\gamma_{12}$ (0.1 *μ*mol/min), $\gamma_{13}$ (∞ or immediately release); NP decay rate ($\lambda_{NP}$) was varied as $\lambda_{NP1}$ (3.2090e-05 1/min), $\lambda_{NP2}$ (9.6270e-05 1/min), $\lambda_{NP3}$ (4.8135e-04 1/min), which represents the half-life time as 15 days, 5 days and 1 day respectively. Finally, the extracellular decay rate λ is 0 in (a) and 1.6045e-5 1/min in (b).

**Fig. 10.**
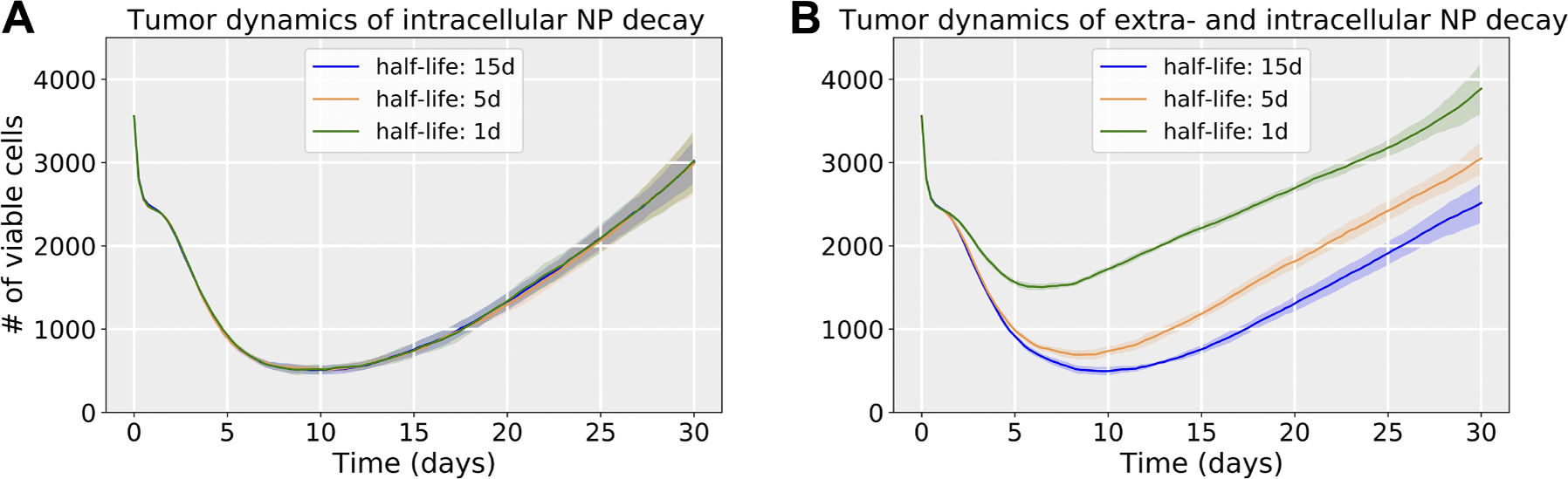
Tumor population dynamics of different NP decay rates for the simulations in Fig. 9 (when drug is *immediately released* by internalized NPs): (a) varying only intracellular NP decay (NP decay rate ($\lambda_{NP}$) was varied as $\lambda_{NP1}$ (3.2090e-05 1/min), $\lambda_{NP2}$ (9.6270e-05 1/min), $\lambda_{NP3}$ (4.8135e-04 1/min), which represents the half-life time as 15 days, 5 days and 1 day respectively); (b) varying both extra- (1.6045e-5 1/min or half-life as 30 days) and intracellular NP decay. Compared with only intracellular decay, increasing both decay rates significantly reduces cancer treatment efficacy even when drug is immediately released. This is because tumor cells would endocytose fewer NPs due to quick decay of extracellular NPs in the microenvironment. Curve and shared areas indicate the mean and confidence interval (defined as ± one standard deviation) over 10 replication runs.

In this exploration, a single NP dose was injected at the beginning of simulation. For the NP decay, we only considered that intracellular NPs can decay. NP inheritance was also ignored in this section, so daughter cells receive zero NPs from their parent after division. Fig. 8 shows that the model is sensitive to the NP internalization rate ($r_{I}$) and drug release rate ($r_{j}$, varied with $\gamma_{1}$ - see Eq. (17)), while it is relatively insensitive to the intracellular NP decay rate ($\lambda_{NP}$), especially in the case of faster drug release, because NPs would release the majority of their drug before they decay. We observed that slow drug release may improve treatments compared to fast release (quantified in terms of viable tumor cells). This is consistent with “adaptive therapy” theory (Gatenby et al., 2009), where *containment treatment* with a limited dose may control cancer growth better than *aggressive treatment*. To explore how intracellular and extracellular NP decay influence treatment results, we performed a 3 by 3 parameter exploration varying both the intracellular and extracellular NP decay (λ in Eq. (5)) rates. Fig. 9 shows that increasing both decay rates would significantly reduce cancer treatment efficacy even when drug is immediately released. This occurs because tumor cells would endocytose fewer NPs due to quick decay of extracellular NP in the microenvironment. See Fig. 10 for tumor population dynamics for the two different scenarios simulated in Fig. 9. The tumor population dynamics show similar tendencies after about 12 days in Fig. 10(b) because only one NP dose was injected at the beginning of simulation. By day 12, the majority of the NPs had already been cleared (e.g., by the renal system) leaving very few NPs to diffuse from the surrounding blood vessels and replace decayed or endocytosed NPs. Note, systemic clear is modeled implicitly as time-varying Dirichlet boundary conditions.

### 3.2. NP therapeutic schedules

**Fig. 11.**
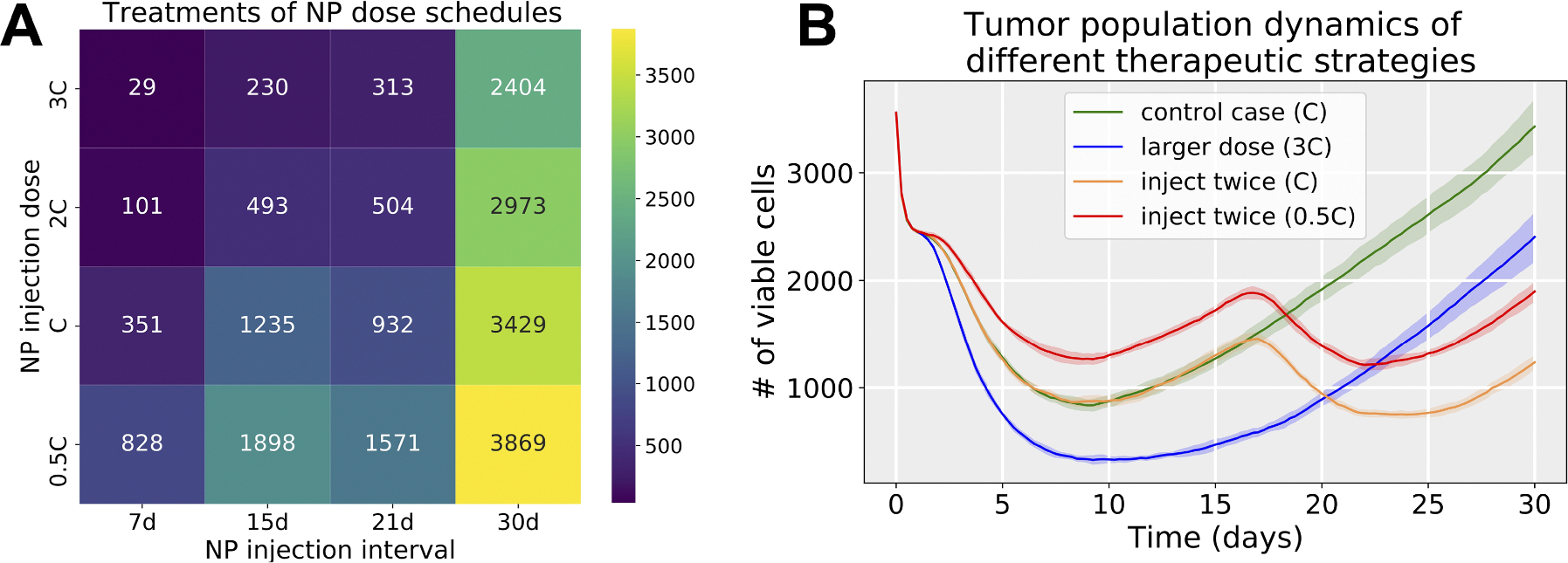
Simulation results of NP injection schedules. (a) Viable tumor cell population heatmap at day 30 (NP internalization rate $r_{I}$: 0.0058 1/min; drug release rate $\gamma_{1}$: 0.1 *μ*mol/min; NP decay rate $\lambda_{NP}$: 9.6270e-05 1/min); (b) Tumor population dynamics of different therapeutic strategies, including a single regular dose (green), a single triple dose (blue), two regular doses (orange), and two half doses (red). Note that C is the default NP injection dose in the simulation. We observe that multiple lower doses may have better performance that single larger doses to control the tumor (e.g., inject twice (0.5C) versus control case (C)). Curve and shared areas indicate the mean and confidence interval (defined as ± one standard deviation) over 10 replication runs.

**Fig. 12.**
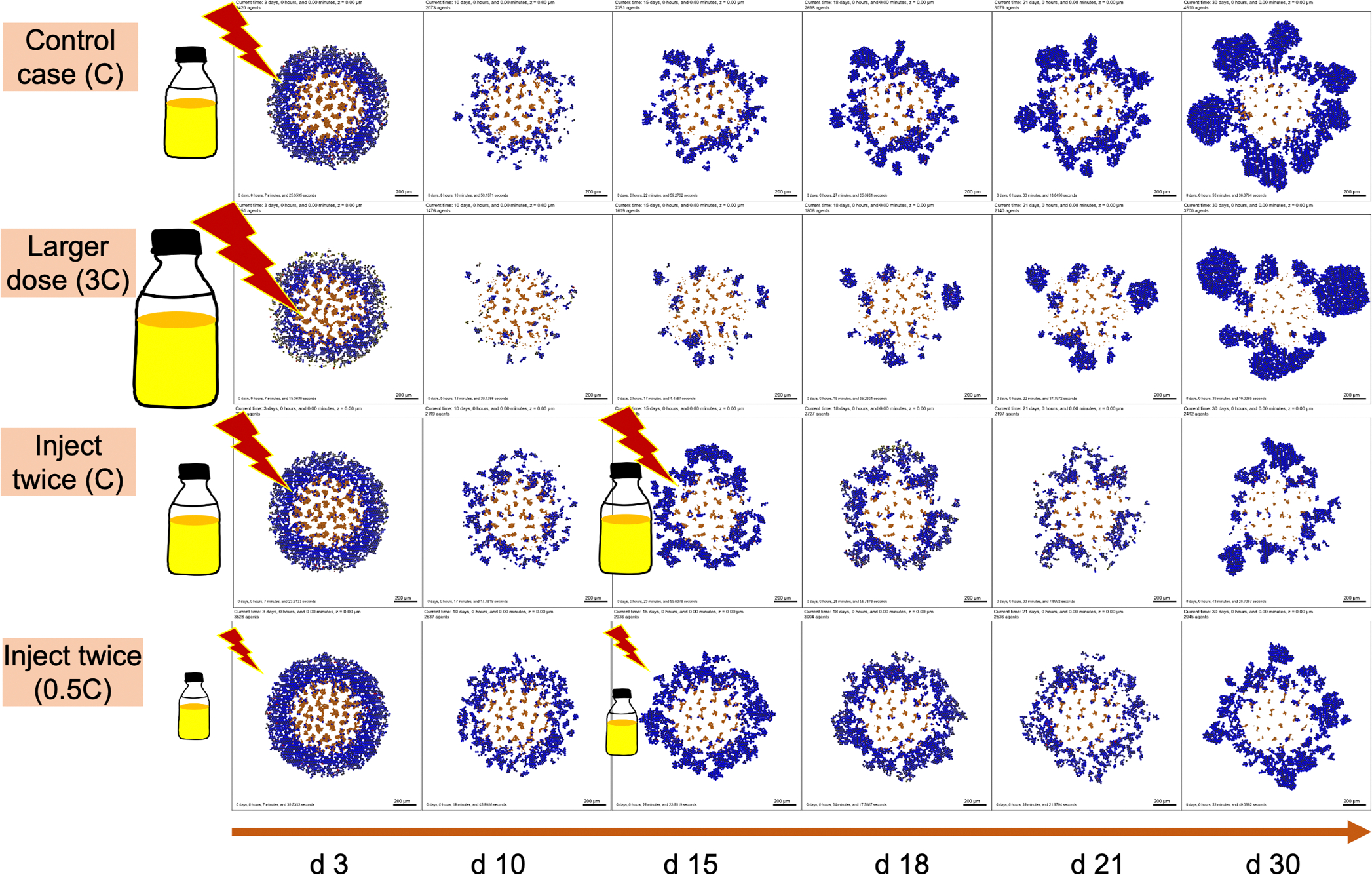
Snapshot of tumor patterns of different therapeutic strategies for the simulation in Fig. 11(b). Cells colored by state. Live cells colored from blue (no drug effect) to yellow (maximum drug effect), brown (necrotic), red (apoptotic). We can observe that the treatment of *inject twice* (0.5C) is better than *control case* (C) even though the total dosage of two scenarios is the same. Additionally, *inject twice* (0.5C) works better than a single large dose (3C) because there are few NPs left in circulation at later stages for a single larger dose.

We next explored NP therapeutic schedules with different NP injection doses and frequencies. We simulated tumor growth dynamics under different treatment strategies (baseline dose to a half-, double-, and triple-dose, and dosing at every 7 days, 15 days, 21 days and once at the beginning.) From Fig. 11(a), we can find that multiple smaller doses may be better than single larger doses (when the total amount of injected NPs is the same). There are several factors that may contribute to this result. (1) Considering the boundary condition as a model of blood vessels/systemic circulation, larger doses do not necessarily ensure that more NPs enter the tumor site from blood vessels, as some are cleared prior to reaching the tumor. (2) Tumor cells need time to endocytose NPs from the microenvironment before they decay. (3) Due to slow NP diffusion in solid tumor tissue, a large dose may not penetrate the tumor from the periphery to the center in a short time, so tumor cells which are far away from periphery would not have access to sufficient NPs for a therapeutic response. (4) From an “adaptive therapy” perspective (Gatenby et al., 2009), higher doses may have decreased efficacy in containing tumor growth compared with lower doses. Because larger doses could quickly kill most cancer cells (in the periphery of the tumor), opening up resources to the interior cells which can proliferate fast. In Fig. 11(b), the second injection does not take effect immediately due to the time delay of NP internalization and drug release. Therefore, optimizing the NP pharmacokinetic design parameters and therapeutic schedule is vital for improving treatment. Fig. 12 gives simulation snapshots of four different therapeutic strategies.

### 3.3. NP inheritance may improve cytotoxic chemotherapy

**Fig. 13.**
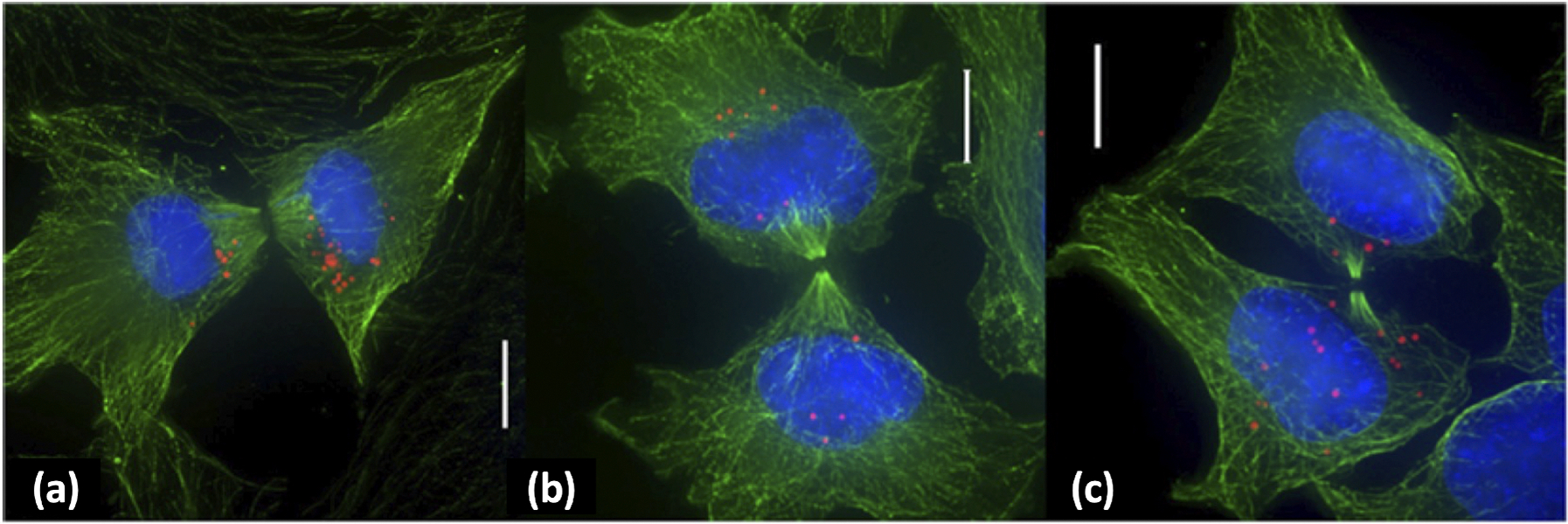
NP inheritance at cell division: Representative deconvolution microscopy images of cells undergoing cytokinesis. Microtubules are stained green, nuclei are labeled blue, and particles are shown in red. Scale bars: 10 *μm*. Adapted with permission from Yan et al. (2013). This is an unofficial adaptation of an article that appeared in an ACS publication. ACS has not endorsed the content of this adaptation or the context of its use.

Experimental work has shown that NPs are potentially inherited by daughter cells during cell division (Yan et al., 2013; Summers et al., 2013); see Fig. 13 for experimental results of NP inheritance during cell division (Yan et al., 2013). Lijster et al. recently used statistical modeling to investigate the impact of asymmetric NP inheritance, finding that the value of the coefficient of variation (standard deviation over mean) of cellular NP counts was greatly impacted by the degree of NP inheritance asymmetry (Lijster and Åberg, 2020).

**Fig. 14.**
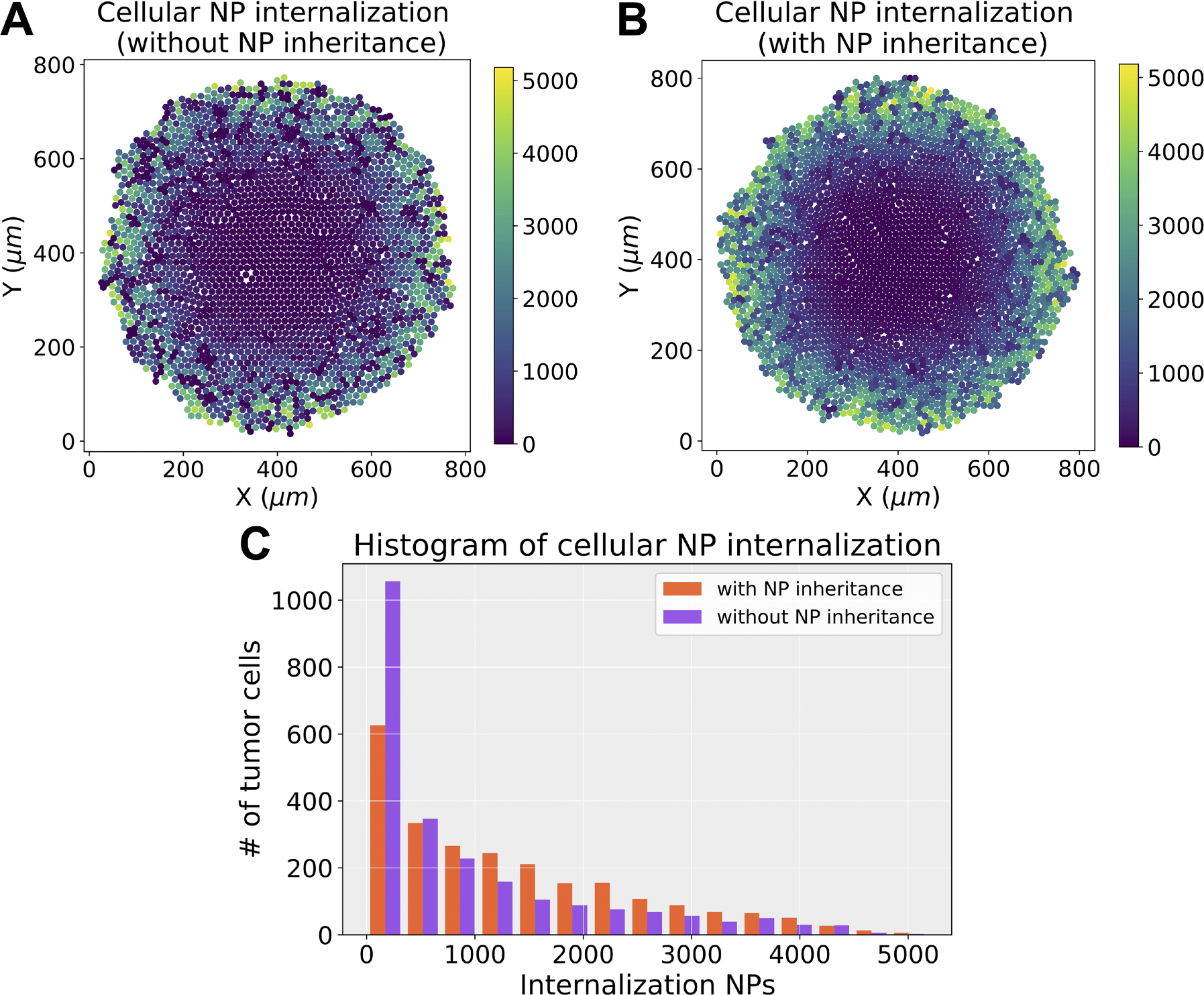
NP inheritance visualization. (a) Cellular NP internalization (without NP inheritance) after 2.5 days of simulation; (b) Cellular NP internalization (with maximum NP inheritance); (c) Comparison of histograms of cellular NP internalization in (a) and (b). We find that tumor cells contain more NPs when they can be inherited at cell division, which raises the possibility of multi-generational treatments with nanotherapy. Note that for illustrative purposes, tumor cells can proliferate (cycle) but cannot die (rates of apoptosis and necrosis set to zero) in the simulations of (a) and (b)). Color bars show number of nanoparticles per cell.

To the best of our knowledge, no mechanistic modeling study has explored cancer nanotherapies where NPs can be inherited at cell division. Fig. 14(a and b) presents simulation results of cellular NP internalization (with and without NP inheritance). Note that we assume a NP’s inheritance is independent of its current drug release state. From Fig. 14(c), we can observe that more tumor cells contain internalized NPs after 2.5 days of simulation if daughter cells inherit NP at cell division, which raises the possibility of multi-generational treatments during nanotherapy. In this section, we explore how this inheritance would affect treatment response, in particular for cytostatic and cytotoxic anticancer drugs.

**Fig. 15.**
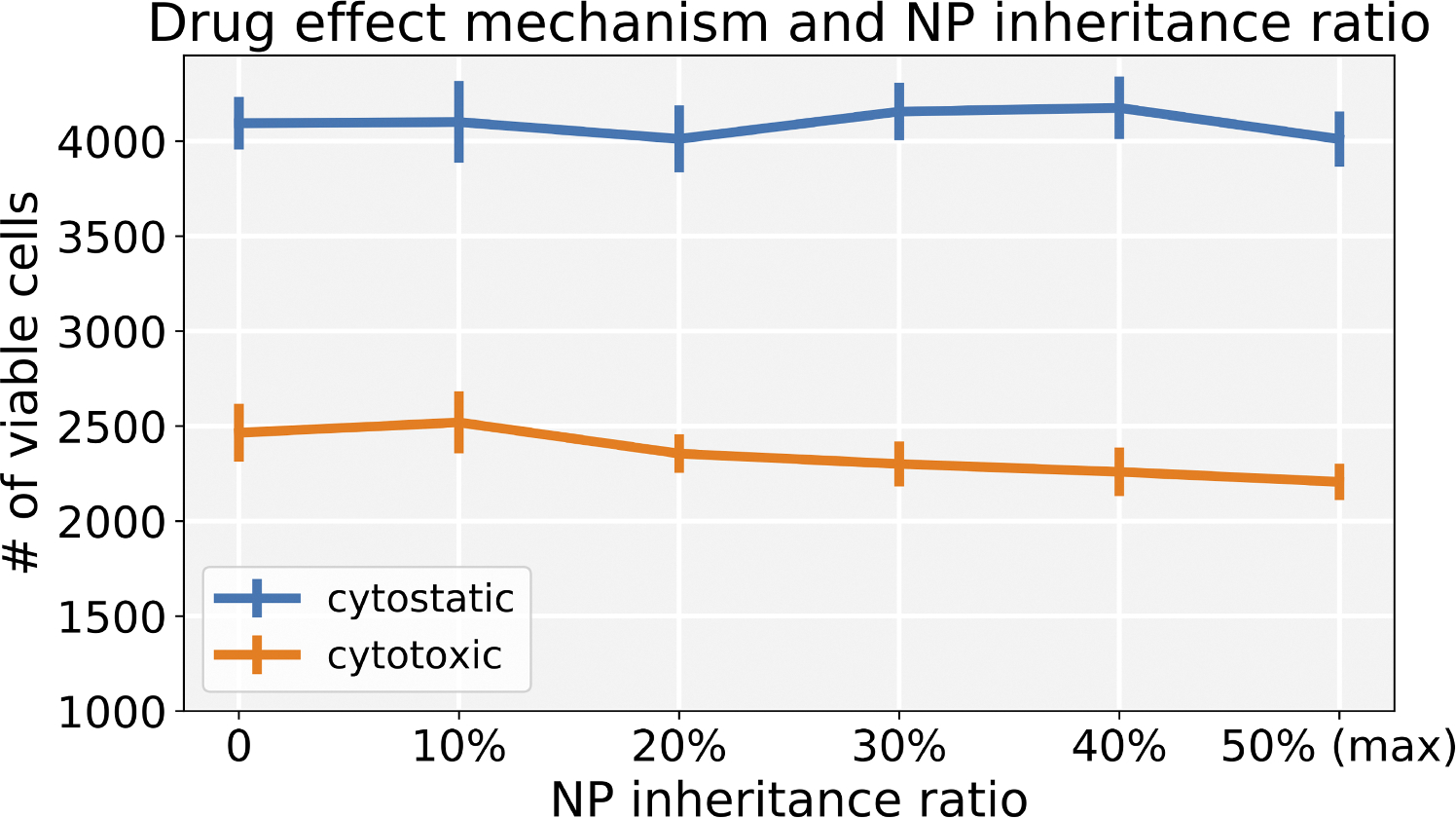
Comparison of cytostatic and cytotoxic drugs treatments across varying NP inheritance ratios (**inject once** (C)). Note that 0 means daughter cells receive zero NPs from parent cell, and 50 % means that each daughter cell gets 50 % of the NPs (maximum). Error bars represents one standard deviation of 10 runs. We can observe that the cytotoxic treatment improves as more NPs are inherited, while there is no clear improvement for cytostatic chemotherapy.

**Fig. 16.**
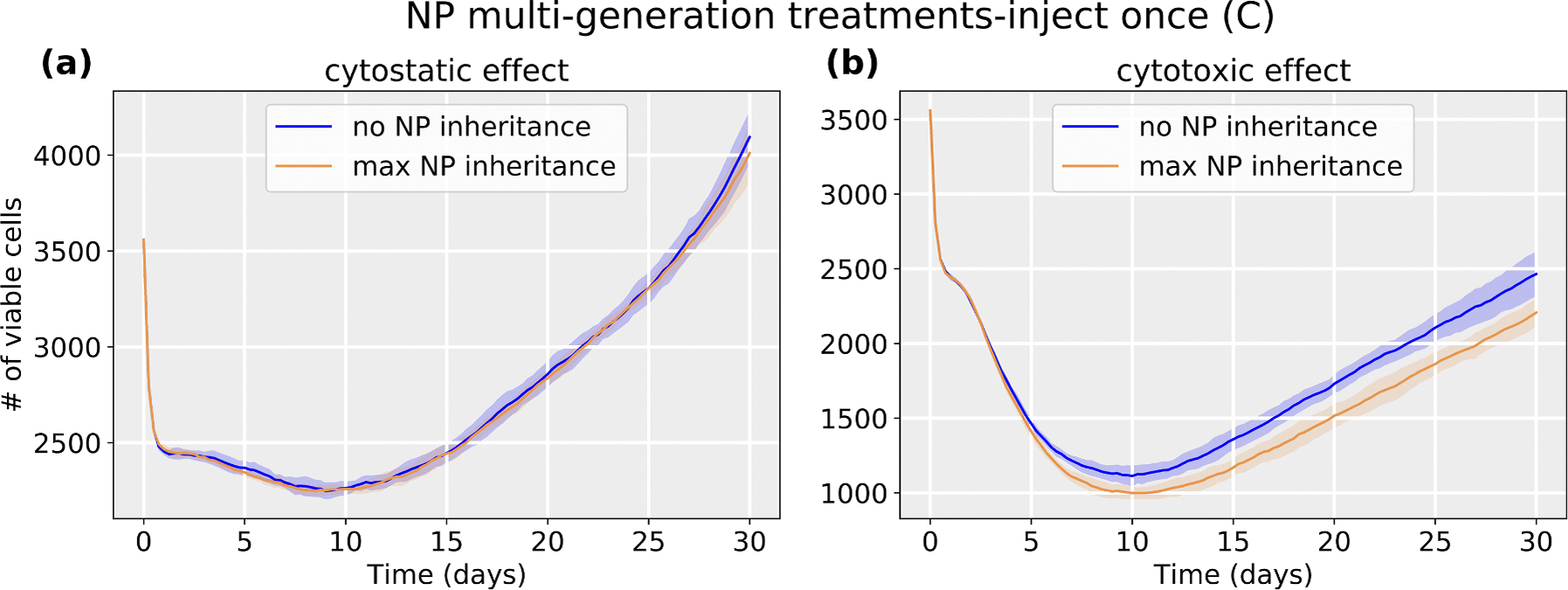
Tumor dynamics of cytostastic and cytotoxic effect treatments at 0 and maximum NP inheritance (**inject once** (C)). Cytostatic drugs inhibit cell cycling, making it difficult to transfer NPs to daughter cells. For cytotoxic drugs, the apoptosis rate is increased. However, there is no effect on the cell cycling rate, so daughter cells can still receive inherited NPs from their parent cell before entering apoptosis. Curve and shared areas indicate the mean and confidence interval (defined as ± one standard deviation) over 10 replication runs.

Varying the inheritance ratio, we first investigated the scenario of injecting a regular dose (C) once at t=0. In Fig. 15, we find that cytotoxic treatment is moderately improved as more NPs are inherited, while there is no clear improvement for cytostatic chemotherapy. Cytostatic drugs inhibit cell cycling, making it difficult to transfer NPs to daughter cells. For cytotoxic drugs, the apoptosis rate is increased, but has no effect on the cell cycling rate. Thus, when treated but not yet apoptosed cells pass their NP to daughter cells at division, therapy can continue to cause additional apoptosis events that slow tumor growth. See Fig. 16.

### 3.4. Cytostatic chemotherapy may be improved by NP inheritance when cell division is not completely inhibited

**Fig. 17.**
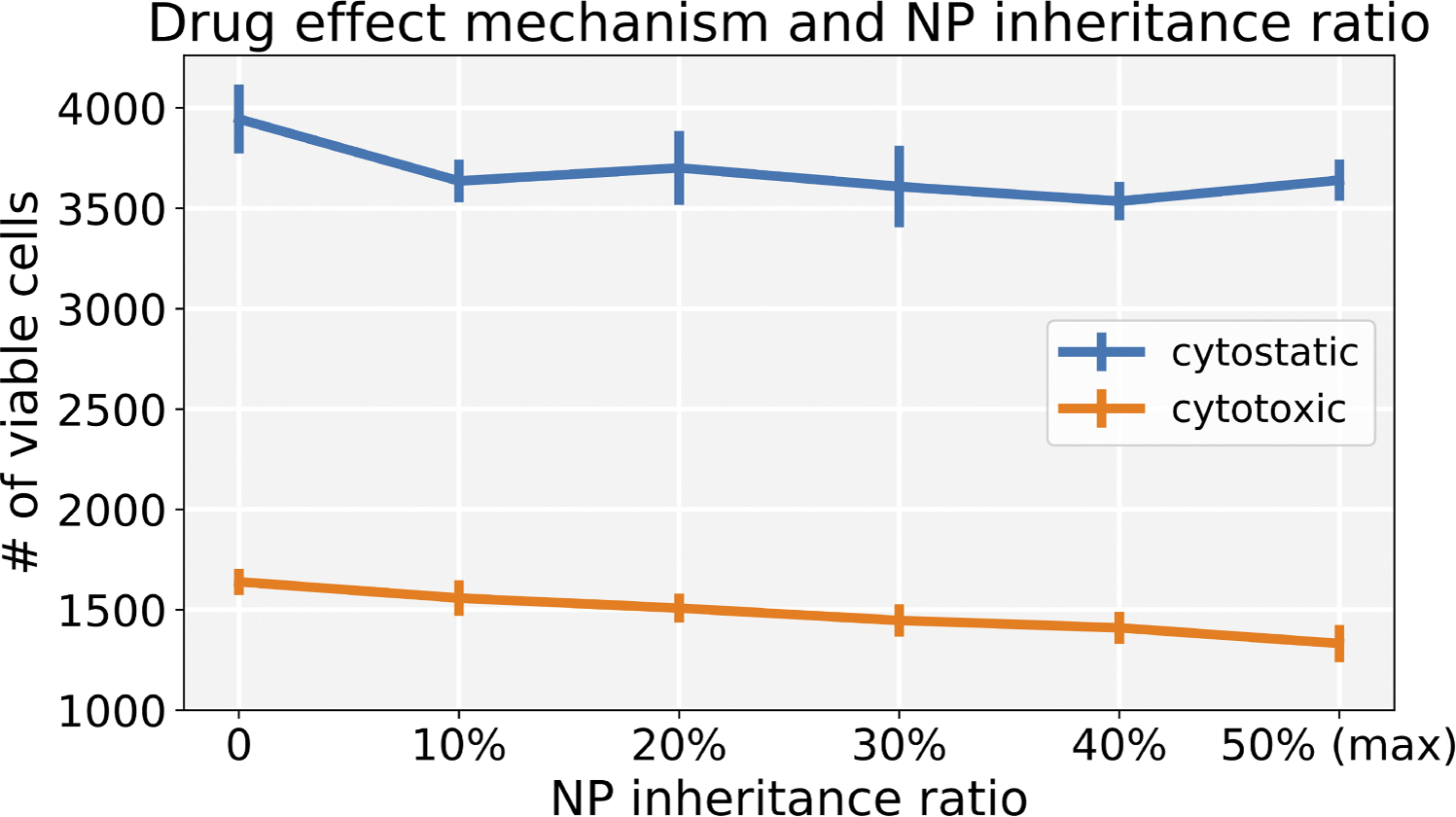
Comparison of cytostastic and cytotoxic drugs treatments with different NP inheritance ratio (**inject twice** (0.5C,0.5C)). Error bars represent one standard deviation of 10 runs. Compared with Fig. 15, we can observe *both* chemotherapies have better response if NP are allowed to be inherited at cell division under two injections.

**Fig. 18.**
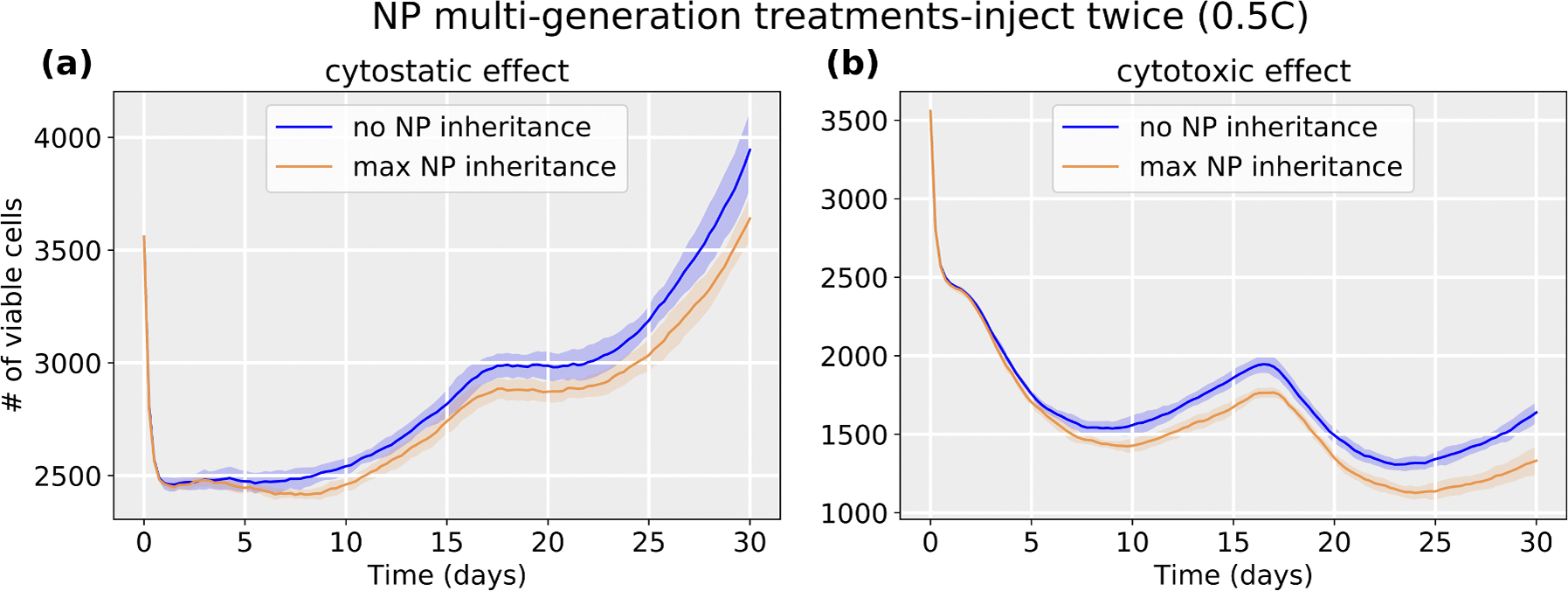
Tumor dynamics of cytostastic and cytotoxic effect treatments at 0 and 50 % NP (maximum) inheritance (**inject twice** (0.5C,0.5C)). We observe that two smaller doses lead to better treatments for both classes of anticancer drugs. Because smaller cytostatic drug doses cannot fully inhibit cell division in a short time (before entering into cycling phase) due to delay of NP internalization, some tumor cells still divide and transfer NP to daughter cells. Curve and shared areas indicate the mean and confidence interval (defined as ± one standard deviation) over 10 replication runs.

We explored another scenario: injecting a half dose twice (0.5C,0.5C) at t=0,15 days respectively. In this case, we found that *both* chemotherapies have better response if NPs are allowed to be inherited at cell division. See Figs. 17 and 18.

## 4. Discussion

NPs offer attractive features for delivery of therapeutic agents to tumor cells, such as improved bio-distribution, protecting therapeutic agents from fast degradation in harsh microenvironments, improved binding rate via functionalized ligands that uniquely interact with receptors on tumor cell membranes, controlled intracellular drug release (via external stimuli, e.g., pH, enzymes, temperature), and enabling cancer immunotherapy through development of synthetic vaccines (e.g., incorporating DNA, siRNA, mRNA and protein) (Sun et al., 2014; Shi et al., 2017; Dogra et al., 2019). In this study, we developed a multicellular framework to evaluate cancer nanotherapy at the single-cell level. The ABM framework includes simulation modules of NP internalization (tracking how many NPs have been endocytosed by each cell); drug release (tracking the drug-release states for each NP as well as how much total drug is retained in individual cells; see Fig. 4); NP inheritance at cell division (see Fig. 14); and pharmacodynamic effects of anticancer drug effects on tumor cell phenotype (e.g., cycling, apoptosis).

In the exploration of pharmacokinetic design parameters, including NP internalization rate, NP decay rate, and NP drug release rate, we found that tumor response is sensitive to the NP internalization rate and drug release rate, while being relatively insensitive to the intracellular NP decay rate, especially in the scenario of faster drug release, because NPs would release most of the loaded drug before they decay (see Fig. 8). The exploration found that slow drug release may improve treatment compared to fast release due to *containment treatment* controlling cancer growth better than *aggressive treatment* with limited dose (based on “adaptive therapy” theory (Gatenby et al., 2009)). Containment treatment aims to maintain a stable tumor burden that restrains “drug-resistant” cells (cells located in the tumor center, with limited access to resources) through competition with “drug-sensitive” cells (cells located in the tumor periphery, with enough available resources), adapting the treatment in real time based on the tumor response, and may also yield better resection outcomes. Furthermore, we varied both intracellular and extracellular NP decay rates to explore their influence on treatment response. We observed that increasing both rates would significantly reduce cancer treatment efficacy even when drug is immediately released (see Figs. 9 and 10), because tumor cells would endocytose fewer NPs due to fast decay of extracellular NP in the microenvironment.

From the exploration of therapeutic schedules modifying different NP injection doses and frequencies, we observed that multiple smaller dosing injections may lead to better treatment outcomes than single larger doses even though the total amount of injected NPs is the same (see Figs. 11 and 12). A closer examination of the simulations suggests an explanation. Because NPs diffuse slowly into the microenvironment and tumor, a single large bolus of NPs may not reach interior tumor cells, whereas a series of smaller doses allows time for outer cells to respond and die, exposing more interior cells to later NP treatment. In addition, some of the NPs may decay before reaching cancer cells far away from the tumor periphery. From an “adaptive therapy” perspective (Gatenby et al., 2009), larger doses may have worse efficacy in containing growth compared to smaller doses due to resource competition among cancer cells (e.g., cancer cells far away from oxygen sources may not have sufficient oxygen to proliferate). Therefore, it may be beneficial to optimize dosing and frequency of cancer nanotherapy taking into account these considerations.

This study highlights the potential of NP inheritance to augment the tumor response. In particular, lower doses of cytostatic therapy had a delay in drug-induced cell cycle arrest compared to larger NP doses. This effect, due to delays in NP internalization, enabled some tumor cells to still divide, transfer NPs to daughter cells, and potentially expose a larger number of cells to the therapy. Interestingly, an almost monotonic decrease in the number of cells is observed for the cytotoxic delivery as the inheritance ratio increases, potentially reflecting the intrinsic binary nature of the cell division.

While this manuscript provided a first investigation on the impact of extensive heterogeneity in the drug release states of internalized nanoparticles (and the first model of heterogeneous populations of nanoparticles within individual cells), the work has several limitations that could drive future exploration. First, we do not model release of drug from NPs as they diffuse through the extracellular space to reach cells. Therefore, NPs may not have a full $C^{*}$ amount of drug when first internalized (and thus begin in population $n_{0}$); future models could include m PDEs for the m release states, with transitions between PDEs similar to our intracellular transition equations. Second, we used a very simple functional form for drug release from internalized NPs ($r_{j}\propto C_{j}$). Future investigations should more deeply explore more mechanistic release rules, such as $r_{j}\propto \left(\beta C_{j}-\frac{C}{V_{\text{cell}}}\right)$, as might be expected if drug release slows as the intracellular drug concentration $\frac{C}{V_{\text{cell}}}$ increases. Similarly, future models could more mechanistically investigate NP endocytosis, potential encapsulation in vesicles (which may reduce their efficacy), and active removal. And as in any theoretical study, future work should seek direct experimental data to drive improved model hypotheses and constitutive relations, and to validate model findings. Because such experiments are costly, early theoretical work as presented here can be important to help motivate interest in the experiments and drive more focused experimental design.

In summary, the proposed nanotherapy model provides a platform for exploring NP design parameters, dosing regimens, and how NP inheritance may impact treatment response. Future work may focus on the NP-receptor binding dynamics (e.g., similar as virion-ACE2 binding (Getz et al., 2021; Risner et al., 2020) and mRNA lipid NP (LNP) and receptor binding (Wang, 2022)), and mRNA vaccine-loaded LNP for cancer immunotherapy (Wang, 2022; Wang et al., 2021b; Stahlberg et al., 2022). With appropriate preclinical and clinical data, this cancer nanotherapy framework could be used to design and calibrate novel patient-specific cancer control strategies. If clinical trials demonstrate that drug-loaded NP can be safely and effectively designed to control or eliminate tumors in individual patients, they could be powerful for use in future cancer patient digital twins (Fertig et al., 2021; Hernandez-Boussard et al., 2021).

## Appendix A. Code availability

Source code for this project hosted on GitHub repository is made public at: https://github.com/MathCancer/PhysiCell-nanobio.

## Appendix B. Primary model parameters

Table B gives the main parameters we used in the model. Unless mentioned otherwise, parameters are at default values for Physicell 1.7.1.

**Table B.1:** Main parameters used in the model.

Parameter	Value	Units

Computational domain size	1600 × 1600 × 20	*μm* × *μm* × *μm*
Computational mesh element size	20 × 20 × 20	*μm* × *μm* × *μm*
Diffusion *dt*	0.01	min
Mechanics *dt*	0.1	min
Phenotype *dt*	6	min

Oxygen diffusion coefficient	100,000 (Ghaffarizadeh et al., 2018)	μm^2^/min
Oxygen decay rate	0.1 (Ghaffarizadeh et al., 2018)	1/min
Oxygen uptake rate	10 (Ghaffarizadeh et al., 2018)	1/min
Oxygen proliferation threshold (σ_1_)	5 (Ghaffarizadeh et al., 2018)	mmHg
Oxygen proliferation saturation (σ_2_)	38 (Ghaffarizadeh et al., 2018)	mmHg
Oxygen necrosis threshold (σ_3_)	5 (Ghaffarizadeh et al., 2018)	mmHg
Oxygen necrosis maximum (σ_4_)	2.5 (Ghaffarizadeh et al., 2018)	mmHg
Oxygen initial condition	38 (Ghaffarizadeh et al., 2018)	mmHg

NP initial condition	0	*μ*mol
Dosing NP (*C*)	2 (assumed)	*μ*mol
NP diffusion coefficient	6 ((He et al., 2019))	*μm*^2^/min
NP decay rate (intracellular, *λ*_NP_)	3.2090e-05, 9.6270e-05, 4.8135e-04 (varied)	1/min
NP decay rate (extracellular)	1.6045e-05	1/min
NP internalization rate (*r*_*I*_)	0.0029, 0.0058, 0.0116 (varied)	1/min
NP clearance rate	4.8135e-04	1/min
Maximum cellular saturated NP (*n**)	6000 ((Chithrani et al., 2006))	dimensionless
Released drug initial condition (*C*_release_)	0	*μ*mol
Conjugated drug per NP (*C**)	100 (assumed)	*μ*mol
Drug release rate (*γ*_1_)	0.02, 0.1, ∞ (varied)	*μ*mol/min
Drug transited states (m)	100 (assumed)	dimensionless
Intracellular drug decay rate (*λ*_drug_)	6.8765e-05	1/min

Drug half-maximum effect (EC_50_)	40 (assumed)	*μ*mol/*μm*^3^
Hill power (n)	1	dimensionless
Cell birth rate under treatment	0	1/min
Cell apoptosis rate under treatment	0.0015	1/min

Initial tumor radius	500	*μm*
Cell maximum proliferation rate $\overset{‾}{b}$	0.00072 (Ghaffarizadeh et al., 2018)	1/min
Cell maximum necrosis death rate ${\overset{‾}{r}}_{\text{nec}}$	0.002778 (Ghaffarizadeh et al., 2018)	1/min
Cell apoptotic death rate	5.3167e-5 (Ghaffarizadeh et al., 2018)	1/min
